# Galectin-3 in prostate cancer and heart diseases: a biomarker for these two frightening pathologies?

**DOI:** 10.1007/s11033-022-08207-1

**Published:** 2022-12-30

**Authors:** Tânia Lima, Luís Perpétuo, Rui Henrique, Margarida Fardilha, Adelino Leite-Moreira, Jose Bastos, Rui Vitorino

**Affiliations:** 1 iBiMED, Department of Medical Sciences, University of Aveiro, Aveiro, Portugal; 2 Departamento de Cirurgia e Fisiologia, Faculdade de Medicina da Universidade do Porto, UnIC, Porto, Portugal; 3 LAQV-REQUIMTE, Departamento de Química, Universidade de Aveiro, Aveiro, Portugal; 4 Department of Chemistry, University of Aveiro, Aveiro, Portugal; 5 Cancer Biology and Epigenetics Group, Research Center of IPO Porto (CI-IPOP) / RISE@CI-IPOP (Health Research Network), Portuguese Oncology Institute of Porto (IPO Porto) / Porto Comprehensive Cancer Center (Porto.CCC), Rua Dr. António Bernardino de Almeida, 4200-072 Porto, Portugal; 6 Department of Pathology, Portuguese Oncology Institute of Porto (IPO Porto), Rua Dr. António Bernardino de Almeida, 4200-072 Porto, Portugal; 7 Department of Pathology and Molecular Immunology, School of Medicine and Biomedical Sciences, University of Porto (ICBAS-UP), Rua de Jorge Viterbo Ferreira, 228, 4050‑313 Porto, Portugal

**Keywords:** Galectin-3, Prostate cancer, Heart diseases, Biomarker, Diagnosis, Prognosis

## Abstract

Galectin-3 (Gal-3) belongs to galectin protein family, a type of β-galactose-binding lectin having more than one evolutionarily conserved domain of carbohydrate recognition. Gal-3 is mainly located in the cytoplasm, but it also enters the nucleus and is secreted into the extracellular environment and biological fluids such as urine, saliva, and serum. It plays an important role in many biological functions, such as angiogenesis, apoptosis, cell differentiation, cell growth, fibrosis, inflammation, host defense, cellular modification, splicing of pre-mRNA, and transformation. Many previous studies have shown that Gal-3 can be used as a diagnostic or prognostic biomarker for heart ailments, kidney diseases, and other major illnesses including cancer. Moreover, it may also play a major role in risk stratification in different diseases, and in this review, we have summarized the potential roles and application of Gal-3 as diagnostic, prognostic, and risk stratifying biomarker from previously reported studies in heart diseases and cancer, with special emphasis on prostate cancer.

## Introduction

Cardiovascular diseases and cancer are leading causes of death that converge at multiple points (molecular mechanisms) and share common risk factors such as obesity, diabetes, hypertension, tobacco, diet, and alcohol, among others [1]. Evidence of this convergence is provided by the cardiovascular peptides Brain Natriuretic Peptide (BNP) and Amino N-terminal pro-BNP (NT-proBNP), two known biomarkers of cardiovascular diseases whose elevated levels have been reported in cancer patients without heart disease and are associated with tumor progression [2]. As a multifunctional protein, Gal-3 has been implicated in cardiovascular diseases, in processes such as myocardial inflammation, fibrosis and remodeling, and in cancer, particularly in cell migration and invasion, inflammation, apoptosis and metastasis [3–5]. However, this protein has not received sufficient attention as a link between these two diseases because it has been studied and reviewed independently for each disease but not for both simultaneously. Therefore, in the present study, the role and applications of Gal-3 as a diagnostic, prognostic, and risk stratifying biomarker in heart disease and cancer, as well as therapeutic approach, were investigated with special emphasis to prostate cancer (PCa).

### Galectin-3 overview

Galectins are carbohydrate-binding proteins with affinity for β-galactoside and contain a conserved carbohydrate-recognition-binding domain (CRD). To date, this family is known to consist of 15 different lectins that are classified into three groups based on the structure of their CRDs. The prototype group, which includes galectin-1, -2, -5, -7, -10, -13, -14, and -15, contains only one CRD. The tandem repeats group (galectin-4, -6, -8, -9, and -12) included two CRDs linked by a non-conserved sequence, and the chimera group, which includes only Gal-3, contains one CRD and one N-terminal domain. Galectins bind the extracellular glycoprotein to cell surface galactosidase via the carbohydrate-binding domain and play important roles in cell growth, adhesion, differentiation, inflammation, and fibrosis [6].

Gal-3, a ∼30-kDa mammalian lectin belonging to the β-galactoside-binding protein family, comprises a short amino terminal domain of 12 amino acids and phosphorylation sites responsible for controlling its nuclear-cytoplasmic translocation [7], a repeated collagen α-like domain that is prone to cleavage by proteases (e.g., PSA and MMPs) and susceptible to phosphorylation at some serines and tyrosines [8], and a C-terminal comprising a CRD with a NWGR anti-death motif conserved in the B-cell lymphoma-2 (Bcl-2) protein family [9].

**Fig. 1:**
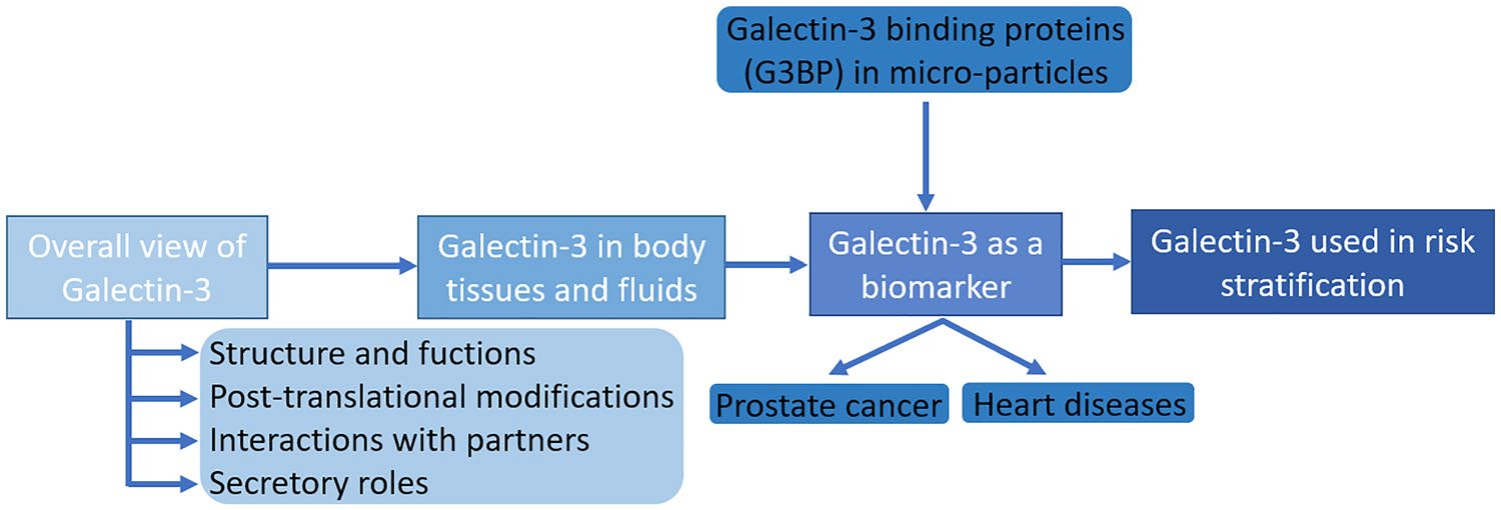
Flowchart of the main topics approached in this review, starting with an overall review of Gal-3 going over points such as: Gal-3 structure and functions, pos-translational modifications, interactions with partners (e.g., protein–protein interactions) and secretory roles. Afterwards, we start to narrow into more and more specific fields: Gal-3 in body tissues and fluids, Gal-3 as a biomarker in several diseases but with more focus on prostate cancer and heart related diseases. At this point we also introduce the concept of micro-particles associated with G3BP. We finish this review with the literature findings about Gal-3 use in risk stratification and what the future holds for this area

Mainly, intracellular Gal-3 is a soluble monomeric protein that can regulate apoptosis and AKT phosphorylation in the cytoplasm [10], as well as transcription, the Wnt/β-catenin pathway, and nuclear pro-mRNA splicing [11, 12]. The extracellular monomer Gal-3 can induce morphogenesis in endothelial cells and angiogenesis in cancer [13]. Due to its unique chimera-like structure, Gal-3 is the only galectin that can form dimers through its N-terminal domain. In addition, it can also form pentamers, which can then cross-link glycosylated ligands and form a dynamic cell surface network or lattice. This may further regulate the presence and endocytosis of glycoproteins and glycolipids in plasma membranes. A flowchart of the major topics covered in this review is shown in Fig. 1.

### Common tissue distribution of Gal-3

Although Gal-3 is found at the cellular level in the cytoplasm and nucleus, this protein can also be secreted into the extracellular environment and into biological fluids (e.g., serum and urine). In the cytoplasm, it plays an important role in cell survival due to its interaction with proteins such as Bcl-2 [9], whose interaction inhibits apoptosis, and guanosine-5’-triphosphate (GTP)-bound K-Ras, as Gal-3 is a mediator of p53 dependent apoptosis [14]. In the nucleus, it promotes mRNA splicing and regulation of gene transcription. In the extracellular environment, the presence of Gal-3 is important for cell–cell and cell–matrix interactions [15].

Gal-3 is integrated into free ribosomes in the cytoplasm, and no signal sequence can be translocated to the endoplasmic reticulum (ER) [16]. It can be transported into the nucleus by passive diffusion and/or active transport. It does not cross the Golgi network or the ER, but there is sufficient evidence that Gal-3 also occurs extracellularly. The protein is secreted by an incompletely understood mechanism, exocytosis, that is not associated with the classical secretion pathway via the Golgi or ER network. Nonetheless, immuno-histochemical studies have shown that the first step of Gal-3 secretion involves its accumulation on the cytoplasmic side of the plasma membrane [16].

**Fig. 2:**
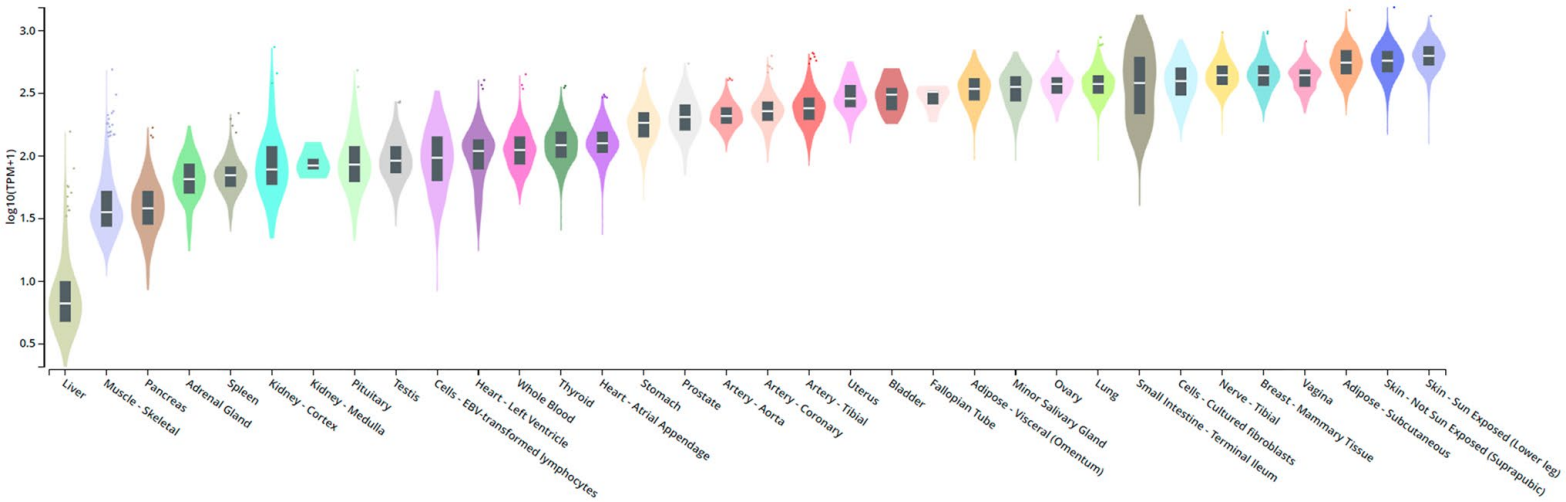
Boxplot of Galectin-3 expression throughout several tissues (in human)

Although the expression of Gal-3 is mainly associated with epithelial cells and myeloid cells, Gal-3 is also found in many other cell types, including skin cells^2^ [17], colonic epithelium^1^ [18] and prostate^1^ [19]. It is also present in salivary glands^1^ [20], pancreas^1,4^ [21], kidneys^3^ [22], intrahepatic bile ducts^1^ [23], fibroblasts^2^ [24], keratinocytes^1^ [25], schwann cells^2^ [26], and gastric mucosa^1^ [27]. The numbers after the cell types (^1^,^2^,^3^,^4^) indicate the experimental models (humans, mice, rats, and hamsters, respectively). There is also a wealth of information on the expression of Gal-3 in cells involved in the immune response, namely neutrophils, eosinophils and mast cells, Langerhans cells, dendritic cells, monocytes, and macrophages from various tissues. In some other cell types, such as lymphocytes, Gal-3 is generally not expressed, although its expression can be triggered by various stimuli [28]. In addition, the pathogenic role of Gal-3 is evident in many tumors of the pancreas, liver, colon, breast, lung, prostate, head and neck, nervous system, and thyroid, leading to the idea that there is a link between the 3 presence and/or absence of Gal-3 and the occurrence of certain cancers. Overall, the tissues/organs/cells with higher Gal-3 secretion are involved in immune system responses and/or have a high impact on metabolism (such as the pancreas, colon, kidneys, and liver). This fact makes Gal-3 an important mediator of processes in the above metabolic pathways, which increases its value as a biomarker in tumors at these secretory sites. The expression of Gal-3 in humans in the various tissues mentioned is shown in Fig. 2, among others.

### Galectin-3 in the clinical setting

Research on the ability of Gal-3 to predict short- and long-term morbidity and mortality has yielded promising results, opening a pathway for the use of Gal-3 in clinical practice. To date, heart failure (HF) studies have been conducted primarily to test the prognostic value of Gal-3 in patients with HF compared with the general population [29]. In these patients, Gal-3 is an important predictor of mortality risk after accounting for age and gender [30]. Based on these findings, a Gal-3 blood test has been proposed for the evaluation of patients with HF [31] and for the management of acute HF patients admitted to the emergency department [3]. Great interest was generated by the Prevention of REnal and Vascular END stage disease (PREVEND) clinical trial, the largest study to date with the longest observation period to investigate Gal-3 levels [32]. A total of 7968 Caucasians from the general population were studied and followed up for 10 years [32]. It is worth noting that serum Gal-3 levels increase with age and women have higher levels on average than men. In this study, there was a clear association between serum Gal-3 level and the increase in mortality due to all previously mentioned causes, suggesting that measurement of serum Gal-3 level can not only effectively predict the prognosis of patients with HF but can also be used in various other pathologies [30, 32].

Patients with HF are frequently readmitted to the hospital after their initial recovery. Detection of Gal-3 in collected body fluids can potentially improve the current management of HF. In addition, knowledge of the value of Gal-3 quantification in the saliva of patients with HF as a tool for stratifying prognosis has been an effective means of reducing rehospitalization rates [33]. HF patients with Gal-3 concentrations greater than 172.58 ng/ml may be at higher risk for future rehospitalization than patients with lower Gal-3 concentrations. If these results are confirmed in a large multicenter clinical trial, measurement of salivary Gal-3 concentrations could be part of the routine evaluation of HF patients [33].

### Interaction with partners

Gal-3 interacts with several partners, but the development of the targets and the functional properties of the galectin-3 binding proteins are not yet clear. One of the interactors is galectin- binding protein (Gal-3BP), also called Mac-2-binding protein. Gal-3BP has been found in a variety of body fluids, including serum, semen, and breast milk [34]. The prognostic significance of serum and tissue levels of Gal-3BP has been found in various cancers, such as breast cancer [35], lung cancer [36], colorectal cancer [37] and PCa [38]. Gal-3 is important in many biological processes, including cell growth, inflammation, apoptosis, remodeling, splicing of pre-mRNA, fibrosis, transformation, differentiation, modification, angiogenesis, and host defense. Previous evidence suggests that Gal-3 may be involved in the pathogenesis of cardiac remodeling [39]. Gal-3 is known to interact with other cellular partners in the cytoplasm and nucleus. Its interaction with the ribonucleoprotein complex was studied by Fritsch et al. [40]. Fritsch and his team found that Gal-3 interacts with the multifunctional ribonucleoprotein particle HnRNPA2B1, a known player in mRNA splicing and in splicing machinery. By knocking down Gal-3, they showed that this protein regulates mRNA export from the nucleus and splicing. The interaction between HnRNPA2B1 and Gal-3 is involved in the early assembly of the splicing machinery and in promoting cell proliferation by modulating the splicing pattern of the oncogene SET [40].

## Gal-3 binding protein (Gal-3BP)

### Biomarker potential of microparticles expressed by Gal-3BP

Gal-3BP is heavily glycosylated and essential for biological processes mediated by galectin. It shows independent and selective binding to the extracellular matrix components of the basement membrane. This protein regulates immunity by upregulating the major class I histocompatibility molecule (MHC-1) [41]. Gene expression of Gal-3BP is particularly pronounced in type I IFN-activated neutrophils and peripheral blood mononuclear cells from patients with systemic lupus erythematosus (SLE) [42]. In a mouse model of venous thrombosis, while Gal-3BP and Gal-3 were detected in platelets, erythrocytes, and circulating microparticles, but Gal-3BP was not present in leukocytes [43]. This underscores the difficulty of translating animal studies of the molecular pathology of thrombosis and SLE to human systems.

### Overexpression of G3BP in microparticles

Overexpression of Gal-3BP in circulating blood microparticles has been observed in SLE patients [44]. An increase in Gal-3BP-related microparticles may be due to increased Gal-3BP load and/or exogenous Gal-3BP binding to the released microparticles. Gal-3BP is overexpressed in microparticles from patients with deep venous thrombosis [45]. It is unclear whether IFN activation leads to an increase in Gal-3BP in microparticles or whether systemic inflammation itself leads to an increase in plasma particles. This underscores the previously mentioned idea that Gal-3BP is one of the few genes that can be strongly induced by IFN [42].

## Post translational modifications of Gal-3

As an oncogenic protein, it is involved in cell growth, differentiation, cell adhesion, chemotherapy resistance, RNA division, apoptosis, and many other physiological and pathological processes related to malignant transformation. Post-translational modifications, such as phosphorylation and cleavage, can effectively regulate the functions of Gal-3 and its interaction with ligands [46]. In response to apoptotic stimuli, Gal-3 migrates from the nucleus to the cytoplasm, a process promoted by phosphorylation. Takenaka and colleagues showed that Ser6 is crucial for this process because when Ser^6^ of Gal-3 is mutated (Ser^6^Ala or Ser^6^Glu), Gal-3 is not phosphorylated or exported from the nucleus. In contrast, wild-type Gal-3 is phosphorylated, exported from the nucleus to the cytoplasm, and protects human breast carcinoma cells from drug-induced apoptosis [46]. Phosphorylation of Gal-3 also regulates its interaction with ligands. The *c-Abl* tyrosine kinase, cyclin-dependent kinase inhibitor (CKI) [47]*,* and glycogen synthase kinase-3β (GSK-3β) [12] can phosphorylate serine (Ser^6^: CKI, Ser^92^ and Ser^96^: GSK-3β) and tyrosine residues of Gal-3 (Tyr^79^, Tyr^107^, and Tyr^118^: c-Abl), thus controlling its localization, cleavage status and associated signal transmission. The main target of the c-Abl proto-oncoprotein is Tyr^107^. Phosphorylation of this residue by c-Abl regulates Gal-3 cleavage by prostate specific antigen (PSA) and consequently angiogenesis, chemotaxis and multivalency [8, 48].

The function of Gal-3 is also regulated by proteolytic processing that destroys its multivalency while retaining its carbohydrate-binding activity. Gal-3 cleavage modulates the bone tumor microenvironment in breast and prostate bone metastasis [49]. Gal-3 cleavage is performed by matrix metalloproteinases (MMPs) [50] and PSA. PSA cleaves Gal-3 between Tyr^107^ -Gly^108^, generating two fragments, one of ~ 30 kDa and the other of ~ 16 kDa, corresponding to intact Gal-3 and cleaved Gal-3 with a functional CRD, respectively [51]. On the other hand, MMPs cleave Gal-3 between Ala^62^-Tyr^63^ and Gly^32^-Ala^33^. This Gal-3 cleavage promotes chemotaxis, invasion, interaction with endothelial cells, and angiogenesis of breast cancer cells [50].

From this information, we can conclude that phosphorylation and cleavage of this protein (Gal-3) plays an important role in altering its function by changing its multivalent properties, localization, and interaction with ligand. As a chimeric lectin, Gal-3 undergoes structural changes mainly through phosphorylation and proteolysis of its non-lectin domains, altering its important biological functions. Phosphorylation of Gal-3 in cells can change its ligand binding from a lectin-carbohydrate to a protein–protein system and have various effects on cellular mechanisms. Phosphorylated Gal-3 induces TNF-induced apoptosis in human breast cancer cells by inducing the expression of *PTEN* (a gene that functions as tumor suppressor) and promoting the sensitivity of TNF-induced apoptosis-inducing ligand (TRAIL) [52]. On the other hand, extracellular Gal-3 causes colon cancer cells to be resistant to TRAIL, by inhibiting TRAIL-based clustering and death receptor endocytosis. The gene encoding Gal-3 (*LGALS3*) is polymorphic in humans and is directly related to the carcinogenic effects of the P64H mutation, TRAIL sensitivity, and proteolytic cleavage of the Gal-3 protein [53].

## Gal-3 in cancer and disease

### Literature discrepancy regarding Gal-3 expression among different types of cancer

Large-scale studies on the expression of Gal-3 in different cancers have been performed using different cell types/tissues and different animal samples [54, 55]. However, controversial results have been reported, which may suggest that the role of this protein depends on the cancer type and tumor stage. Indeed, Gal-3 seems to act as a tumor suppressor in some cancers such as endometrial [56], prostate [4] and melanoma [17] cancers*,* as its levels are reduced in cancer patients compared to controls. In other cancers such as hepatocellular cancer [57], pancreatic cancer [21]*,* colon cancer [18]*,* gastric cancer [27]*,* bladder cancer [58]*,* lung cancer [59]*,* thyroid cancer [60], it appears to play a tumor-promoting role as its levels are increased in these cancer patients compared to non-cancer patients and promote cancer progression. In other cases, such as renal cell carcinoma and breast cancer, increased [59] and decreased [22] Gal-3 levels have been detected in the serum and tumor tissues of these patients. In addition to expression, the cellular distribution of Gal-3 also appears to vary by cancer type. In prostate, breast and colon cancer cells, translocation of Gal-3 from the nucleus to the cytoplasm is observed, suggesting antitumor or pro-oncogenic activity of this protein, depending on whether it is localized in the nucleus or cytoplasm [61–63]*.*

The specific experimental techniques, including the antibodies used, Gal-3 localization, the inclusion of appropriate controls, and the diversity of the tissues studied may partially explain the differences between studies. In general, histological techniques that allow cell-specific analysis of Gal-3 expression, such as immunohistochemistry or studies using in situ hybridization, are more reliable than RT-PCR and Western blot-based methods because they may lead to impurities or diluted results (noncancerous cells may also be present). The molecular functions of Gal-3 and its contribution to tumors remains unclear, but data suggest that different concentrations, distributions, and locations are characteristic and specific for certain cancers. It remains to be seen whether this can be used as a fingerprint for these cancers. However, most researchers agree that Gal-3 detection can contribute to improve the diagnosis and prognosis of human cancer [61].

### Gal-3 in prostate cancer

Having contextualized the role of Gal-3 in different cancers, we focus on PCa, one of the most common cancers in men [64]. The literature on the association between Gal-3 and PCa was reviewed, and a summary of the studies, including the type of sample used, the number of patients included, and the main findings, is presented in Table 1. From these studies, it appears that malignant transformation of the prostate is associated with cellular redistribution of Gal-3 and a decrease in tissue levels of this protein compared with normal prostate, which correlates with PCa progression, and resistance to apoptosis. Indeed, in PCa cells, a shuttling between the nucleus and cytoplasm is observed, with Gal-3 translocating from the nucleus to the cytoplasm [61]*.* In the cytoplasm, Gal-3 promotes tumor growth, angiogenesis, and resistance to apoptosis, whereas in the nucleus it produces the opposite effects and exhibits an anti-apoptotic effect [65]. It seems that the anti-apoptotic effects of cytoplasmic Gal-3 are related to the induction of phosphorylation and reduction of Bad (pro-apoptotic protein) expression, which is responsible for regulating the expression of Bcl-2, and the consequent stabilization of mitochondrial membrane potential. In this way, cytochrome C is not released from mitochondria, caspase-3 is not activated, and prostate tumor cells do not undergo apoptosis [66]. The decreased Gal-3 levels in prostate tumor tissue could be due to cleavage and/or hypermethylation of the gene promoter. As mentioned earlier, proteolytic processing of Gal-3 occurs in the collagen-like linker region in PCa. Cleavage of Gal-3 occurs exclusively in tumor tissue and is not observed in normal prostate. Progression of PCa is associated with an increase in cleaved Gal-3 and a decrease in intact Gal-3, which promotes tumor progression, malignancy, and metastatic process. The intact Gal-3 can be detected with an antibody against the N-terminal domain, whereas the cleaved form can be detected with an antibody against the C-terminal fragment of Gal-3. It was suggested that the cleaved Gal-3 is mainly localized in the cytoplasm [5]. Ahmed et al. investigated the methylation status of the Gal-3 promoter and found that it was highly methylated in malignant prostate epithelial cells, human PCa tissue, and serum samples, but not in normal cells, prostate tissue, and serum samples from BPH patients. However, the degree of methylation varied by disease stage, with early stages (I and II) showing strong methylation and advanced stages (III and IV) showing mild methylation. This methylation profile is important for the early diagnosis of PCa. In controls, the Gal-3 promoter was virtually unmethylated in normal prostate and BPH samples [67, 68]*.* From a non-invasive point of view, it is important to note that although these are unpublished data, Ahmed and his team claim that methylation of the Gal-3 promoter was detected in all urine samples from PCa patients, but not in urine samples from kidney and bladder cancer patients [67]. Since the bladder and kidney contribute significantly to the composition of urine, these results may be promising for a specific and non-invasive diagnosis of PCa by urine analysis.

**Table 1:** Studies regarding the role of Galectin-3 in Prostate Cancer

Study	Type of sample	Number of patients enrolled	Main findings
Li et al. [74]	Prostate tissue	N = 57, including Gleason pattern 3 (tumors of 3 + 3), 4 (tumors of 3 + 4, 4 + 3, or 4 + 4) and 5 (tumors of 5 + 4 or 4 + 5)	Gal-3 decreased from non-aggressive tumors to Gleason pattern 5 tumors. Galectin-3 showed a specificity of 89% and a sensitivity of 36.3% for distinguishing aggressive from non-aggressive tumours
Pacis et al. [19]	Prostate tissue	PCa tissue samples (n = 39); Matched paraffinized normal prostate tissues (n = 39)	Gal-3 is downregulated in PCa
Gao et al. [5]	Prostate tissue	PCa (n = 66): GS6-7 (n = 43); GS8-9 (n = 23); Stage I-II (n = 29); Stage III-IV (n = 37) BPH (n = 73)	Gal-3 cleavage occurred in PCa but not in normal prostate. Increased cleaved Gal-3 levels but decreased intact Gal-3 levels during PCa progression Cleaved Gal-3 as diagnostic biomarker and therapeutic target for PCa. It is suggested that PSA mediates the degradation of intact Gal-3. Cleaved Gal-3 was mostly localized in cytoplasm
Wang et al. [75]	Prostate tissue Prostate cell lines	Normal prostate tissue (n = 8) Malignant tumor tissue (n = 40)	Increased Gal-3^+^/ AR^+^ cells in patient tissues as a new marker for distinguish PCa subtypes and to guide personalized treatment
Ellerhorst et al., 1999 [76]	Prostate tissue	Normal human prostate (n = 8) PIN tissues (n = 8) Primary prostate adenocarcinomas (n = 20) PCa metastases (n = 12)	Decreased Gal-3 levels in primary carcinoma and metastatic disease compared with normal and premalignant tissue Gal-3 as biomarker of PCa progression
Ellerhorst et al. [76]	Prostate tissue	Normal prostate tissues (n = 15) Adenocarcinoma prostate tissues (n = 15) BPH tissues (n = 15)	Reduced Gal-3 tissue expression in adenocarcinoma compared to normal tissue. Increased Gal-3 tissue expression in BPH when compared with the adenocarcinoma and normal prostate
Araújo-Filho et al. [77]	Prostate tissue	145 prostate samples from clinically localized PCa	Reduced Gal-3 tissue expression in PCa compared with normal glands. In PCa, Gal-3 was excluded from the nucleus and was only present in cytoplasm Significant association between PSA relapse and Gal-3 expression Biomarker of tumor progression
Brûle et al. [61]	Prostate tissue	Hormone-refractory tissue (n = 95) Benign tissue samples (n = 150) Adjacent bening tissue (n = 300) Primary tumor tissue (n = 129)	Decreased Gal-3 expression in the primary PCa. specimens and in metastatic lesions compared with benign or pre-malignant tissue samples Loss of Gal-3 from a hormone-sensitive to a hormone-insensitive PCa
Merseburger et al. [4]	Prostate tissue Prostate cell lines	Normal prostate tissues (n = 30); PIN (n = 30); GS3 and 4 (n = 82); Metastatic PCa (n = 26); Human PCa cells (PC3 cells)	Gal-3 knockdown in PC3 cells led to cell-cycle arrest at G1phase Gal-3 cleavage occurred at GS3 and GS4, but not at normal and PIN tissues Gal-3 is cleaved during PCa progression. Cleaved and intact Gal-3 are a diagnostic marker of PCa
Wang et al. [78]	Prostate cell lines Prostate tissue	Normal prostate epithelial cells (PrEC) Malignant prostate epithelial cells (LNCaP) Benign prostate epithelial cells (BPH-1) Normal and tumor prostate tissue	Gal-3 poorly or not expressed in BPH-1 and LNCaP cells compared to PrEC Gal-3 expression is regulated by promoter methylation in LNCaP cells and human prostate tumor tissues Gal-3 promoter was highly methylated in human PCa tissue but not in normal tissue Early diagnosis of PCa
Ahmed et al. [68]	Prostate tissue	Tissue microarrays from 83 patients undergoing prostatectomy for clinically localized PCa Tumor prostate tissue (n = 83); Benign tissue (n = 75); adjacent benign prostate tissue (n = 78)	Gal-3 showed nuclear and cytoplasmic localization in benign, adjacent-benign and tumor tissues Decreased Gal-3 staining scores from benign to adjacent-benign and to tumor tissues Gal-3 expression in tumor significantly correlated with biochemical recurrence Gal-3 staining in tumor tissues had 91.7% sensitivity, 64% specificity and73% accuracy in predicting biochemical recurrence
Knapp et al. [79]	Blood Prostate tissue	Metastatic PCa (n = 8); Non-cancer patient controls (n = 8)	Increased serum Gal-3 levels in metastatic PCa when compared with non-cancer controls Gal-3 can be found in both normal and cancer tissue More cleaved Gal-3 in normal tissue. The amount of cleaved and intact Gal-3 in PCa tissue is different from case to case Prognostic/Diagnostic biomarker Serum complementary marker to the PSA blood test
Balan et al. [70]	Blood	N = 95 patients: healthy controls (n = 19), newly diagnosed patients (n = 19), no recurrence after local therapy (n = 19), rising PSA after local therapy (n = 19), and metastatic patients (n = 19)	No significant difference in serum Gal-3 levels across the groups A positive association between Gal-3 and PSA levels among all 95 men was found
Nakajima et al. [80]	Prostate tissue Blood	**Prostate tissue:** Prostate adenocarcinoma (n = 25); BPH (n = 20); normal prostate tissue (n = 16) **Serum samples:** Prostatic adenocarcinoma (n = 18); BPH (n = 15); healthy individuals (n = 10)	Increased cytoplasmatic Gal-3 protein expression in BPH compared with normal prostate tissue Decreased cytoplasmatic Gal-3 protein expression in PCa compared with normal prostate (BPH > healthy > PCa) Decreased serum Gal-3 levels in PCa and BPH compared with healthy individuals
Melo-Júnio et al. [69]	Urine Tissue	N = 23 urine samples (9 PCa patients without relapse + 7 PCa patients with relapse + 7 control samples). Control samples: n = 4 Bladder cancer patients; n = 3 controls without cancer) N = 37 prostate tissue samples (prostatectomy specimens without relapse- n = 12; cancer samples with relapse n = 11; tumor free prostate samples n = 14)	Decreased urinary and tissue Gal-3 levels in PCa patients with biochemical relapse compared with patients without relapse Biomarker of prediction of PCa progression
Geisler et al. [72]	Urine	PCa patients (n = 30); controls (n = 30)	Decrease in urinary Gal-3 protein levels in PCa patients compared with noncancer patients
Lima et al. [73]	Prostate tissue Prostate cell lines Urine Blood	Normal prostate tissue (n = 5) BPH tissue (n = 2) PCa tumor prostate tissue (n = 27): Stage I (n = 11); Stage II (n = 7); Stage III (n = 7); Stage IV (n = 2) PCa cell lines (LNCaP, C-3, DU-145) Serum samples: BPH (n = 1); PCa stage II (n = 2); PCa stage III (n = 1); PCa stage IV (n = 1)	Decreased Gal-3 tissue expression in more advanced stages (mostly in cytoplasm) Gal-3 detection in nucleus and cytoplasm in normal, BPH, HGPIN and stage I tissues, in the later stages (II, III, and IV) of PCa, it was mostly found in the cytoplasm Increased Gal-3 promoter methylation as disease progresses Gal-3 promoter is completely methylated in stages I and II PCa Gal-3 promoter in normal and BPH tissues is almost unmethylated Gal-3 promoter methylation in all urine specimens (22/22) and for all stage II serum samples (2/2), but not methylated for BPH (1/1), stage III (1/1), and stage IV (1/1) serum samples

*BPH* Benign prostatic hyperplasia*; PIN* Prostatic intraepithelial neoplasia

While a decrease in Gal-3 concentration has been consistently found in prostate tissue from PCa patients, the same is not true for blood samples from these patients. Indeed, decreased [69], increased [70], or unchanged [71] serum Gal-3 levels have been found in PCa patients compared with controls. As for the least invasive sample, to our knowledge, only two studies have investigated urinary Gal-3 levels in PCa patients. One study focused on the prognostic utility of urinary Gal-3 levels and reported reduced urinary Gal-3 levels in PCa patients with biochemical relapse compared with patients without relapse [72]*.* The other study focused on the diagnostic potential of urinary Gal-3 protein levels in PCa patients and found that urinary levels of this protein were reduced in PCa patients compared with noncancer patients [73]. To our knowledge, urinary Gal-3 levels in RCC patients have not been investigated, and only one study examined this protein in urine samples from bladder cancer patients, suggesting that it may be a distinguishing feature between bladder cancer patients and control subjects. The only results comparing Gal-3 in these three cancers are those previously reported, in which different patterns of Gal-3 promoter methylation were found [67]. For specific and non-invasive detection of PCa, further studies should be conducted to detect these three cancers in urine by determining Gal-3.

### Gal-3 as a potential marker of heart diseases

Although constitutive expression of Gal-3 in the heart is low, heart diseases such as heart failure are associated with higher levels of Gal-3, although it is unclear whether such mechanisms are already activated when hypertrophied hearts still appear well compensated [81]. The first study on the role of Gal-3 in heart failure was published by Sharma et al., in 2004 [81]. The authors studied severe hypertension and heart failure in a rat model and found that Gal-3 is a highly regulated gene that is strongly overexpressed in decompensated heart. In the mice with heart malformations, the expression of Gal-3 was more than five times higher than in the compensated hearts of controls. In the same study, these results were also applied to human heart tissue and confirmed. Gal-3 exposure analysis was performed on ventricular biopsies from aortic stenosis patients, comparing cases with preserved and reduced ejection fraction, and upregulation was found in myocardial biopsies from patients with reduced ejection fraction [81]. Experimental studies have shown that Gal-3 is the cause of left ventricular remodeling. For this study, mouse models were used in which Gal-3 was infused into the pericardium [82]. The structural and functional changes of rat myocardial tissue can be inhibited by the simultaneous administration of natural N-acetyl-seryl-aspartyl-lysyl-proline (Ac-SDKP), which can be used as a Gal-3 inhibitor. The mechanism of Gal-3 at the myocardial level of cardiac muscle has not been fully elucidated [82]. Once overexpression of Gal-3 is observed, it is responsible for the activation of fibroblasts and macrophages, leading to fibrosis, scarring, and eventual remodeling of cardiac tissue. Gal-3 seems to be involved in the regulation of interstitial fibrosis, especially in the case of cardiac overload. Confirmation of the role of myocardial Gal-3 expression in patients with HF has led some investigators to explore whether serum Gal-3 levels are an effective tool for diagnosing acute HF with dyspnea [83]. The clinical application of Gal-3 in the diagnosis of HF has not yet been clearly demonstrated. Indeed, Gal-3 levels are not related to the New York Heart Association (NYHA) functional classification, which relates the patient´s clinical signs and symptoms to the severity of heart failure. Moreover, the specificity and sensitivity to identify HF cases is low compared to NT-proBNP [83]. Nevertheless, Gal-3 could be an effective marker for risk stratification of HF patients. Interestingly, low serum Gal-3 levels (< 19 ng/mL)are associated with better survival and lower HF rates in patients treated with rosuvastatin [84]. Further evidence of the clinical benefit of Gal-3 in the treatment stratum comes from the multicenter Automated Defibrillator Implantation in Cardiac Resynchronization Therapy trial (MADIT-CRT) [85]. Patients with higher basal levels of Gal-3 benefited more from cardiac resuscitation (65% risk reduction) than patients with lower basal levels of Gal-3 (25% reduction in risk) [85]. The clinical applicability of serum Gal-3 measurements in subsequent treatments was investigated in patients with a left ventricular assist device (LVAT) [86]. However, in these patients, the heart had no effect on plasma Gal-3 concentrations within the first 30 days after implantation. Therefore, Gal-3 may not provide sufficient discrimination to predict outcome [86, 87].

**Fig. 3:**
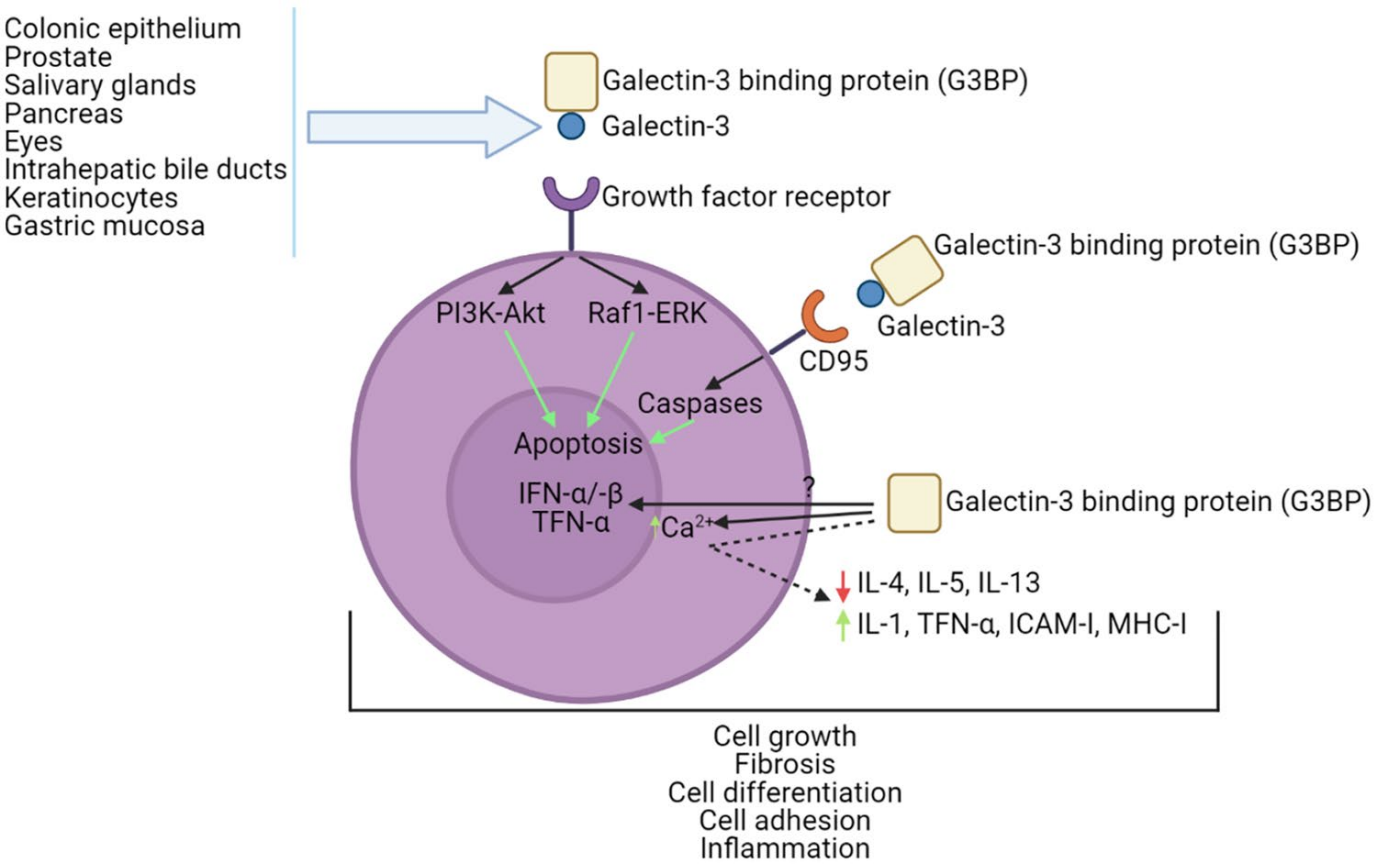
Tissues and organs such as colonic epithelium, prostate, salivary glands, pancreas, eyes, intrahepatic bile ducts, keratinocytes, and the gastric mucosa (among others) are known for their Gal-3 expression. In this image it is shown how Gal-3 + G3BP interact with several different receptors (GFR, CD95, etc.) and a very summarized cascade pathway that occurs in the cellular system. These events lead to phenomena, i.e., biological functions that are regulated by Gal-3, such as apoptosis, cell differentiation, cell growth, fibrosis, inflammation, and cell adhesion (among others)

In summary, Gal-3 is a protein that in humans is encoded by the *LGALS3* gene. Several of the studies mentioned above demonstrate the biological involvement of this protein in cancer, fibrosis, heart disease, and stroke, as well as in processes such as cell growth, cell adhesion, and inflammation. In humans, some of the tissues/organs in which Gal-3 secretion is relevant are the colonic epithelium, gastric mucosa, salivary glands, pancreas, eyes, and prostate (Fig. 3). Studies in patients with previous HF episodes have found a correlation between increased Gal-3 and greater risk of re-hospitalization. Table 2 provides a summary of clinical studies of Gal-3 in patients with heart disease, the type of each sample, and the main findings.

**Table 2:** Studies regarding the role of Galectin-3 in heart diseases

Study	Type of sample	Number of patients enrolled	Main findings
Erkilet et al. [87]	Myocardial tissue (HF)	175 patients received a ventricular assist device (VAD)	Plasma Gal-3 levels are associated with severe HF but do not provide enough information to allow outcome prediction after VAD implantation
Gagno et al. [88]	Myocardial infarction	n = 496 of patients that survived acute myocardial infarction (AMI)	The assessment of Gal-3 and G3BP could aid in risk stratification after AMI
Gullestad et al. [84]	HF	n = 1492 patients with ischaemic systolic HF that were assigned rosuvastatin or a placebo	A treatment with rosuvastatin was beneficial for patients with ischaemic systolic HF with Gal-3 levels below 19.0 ng/mL
Ho et al. [32]	HF	n = 3353 of patients, n = 166 developed HF	Elevated levels of Gal-3 were associated with increased risk of HF and mortality
Lok et al. [30]	HF	n = 232 patients with chronic HF	Plasma Gal-3 is a biomarker for patients with chronic HF, with its prognostic value being independent of the severity of HF. Therefore, it can be used in the management of those patients
Stolen et al. [85]	HF	n = 654 NYHA functional class I/II patients	Gal-3 elevated levels was found to be an independent predictor of adverse outcomes related to HF in patients with mild HF
van Kimmenade et al. [83]	HF	n = 599 presenting dyspnea, of which n = 209 had acute HF	Gal-3 presented as a useful biomarker for evaluation of patients with suspicion or proven acute HF. The combination of Gal-3 with NT-proBNP was the best predictor for prognosis in patients with acute HF
Medvedeva et al. [89]	HF	190 patients divided into 3 groups based on their NYHA functional class	Higher Gal-3 levels in patients with chronic HF and shows positive correlation with oxidative stress and inflammation markers. Gal-3 is a predictor of mortality in patients with chronic HF
Meijers et al. [90]	HF	n = 902 patients divided in 3 cohorts (COACH, n = 592; PRIDE, n = 181; and UMD H-23258, n = 129)	Plasma Gal-3 is a predictor of near-term rehospitalization and fatal event on postdischarge
Anand et al. [91]	HF	Starting cohort of n = 1650, after 4 months n = 1346, after 12 months n = 1097	Patients with HF show elevated levels of Gal-3, with highlight for those with severe HF and renal dysfunction. The overtime increase of this cohort was independently associated with worse outcomes
Polat et al. [92]	HF	n = 44 patients, n = 38 controls	Gal-3 is a biomarker for the diagnosis of patients with HF with preserved ejection fraction
Maiolino et al. [93]	CHD	n = 1013 of randomly selected patients who underwent coronary angiography and long-term follow-up	Gal-3 is a strong independent predictor of cardiovascular death in high cardiovascular risk patients referred for coronary angiography

*HF* Heart failure; *CHD-* Coronary heart disease; *NYHA* New York Heart Association; *COACH* Comparison of Outcomes and Access to Care for Heart Failure; *PRIDE* Proteomics IDEntifications database; *UMD H-23258* University of Maryland Pro-BNP for Diagnosis and Prognosis in Patients Presenting with Dyspnea study, *NT proBNP*- The N-terminal portion of the B-type natriuretic peptide

### Gal-3 potential as a therapeutic target

In this document, we have mentioned several diseases and conditions in which Gal-3 plays a role or appears to affect biological functions leading to certain conditions. Studies have shown that Gal-3 in combination with other compounds such as RN1 (a polysaccharide), TFD100 (a glycopeptide from cod), galectin-3C (a truncated form of Gal-3 that contains the carbohydrate binding domain but lacks some amino acids at the N-terminus) and modified citrus pectin (MCP) (among others) leads to improvements in several Gal-3-related diseases. RN1 is a polysaccharide that suppresses the expression of Gal-3 by binding to it, resulting in growth inhibition in pancreatic ductal adenocarcinoma [94]. TFD100 is a glycopeptide that blocks Gal-3 mediated angiogenesis and metastasis of PCa cells in mice [95]. Galectin-3C is a dominant-negative form of Gal-3 and it is thought to act by blocking endogenous Gal-3 and may be a potential treatment for multiple myeloma [96]. Gal-3C also shows improvements in ovarian cancer patients by reducing the growth, invasion, motility, and angiogenic potential of ovarian cancer cells in these patients [97]. Finally, MCP is a Gal-3 inhibitor that reduces the viability of prostate carcinoma cells and sensitizes them to radiotherapy. MCP in combination with radiotherapy promotes downregulation of Gal-3, impairment of DNA repair machinery, and increase in reactive oxygen species (ROS) production [98]. These studies show that Gal-3 plays an important role in several diseases and its manipulation has a great impact on ameliorating the effects and isa promising therapeutic target. The development of new targets for this protein could be s starting point for the therapy of numerous diseases. Although high levels of Gal-3 are associated with poor prognosis, the association of this protein with other substances could be a target for therapy of some diseases and cancers.

### Gal-3 for risk stratification

The classification of a patient population into low, intermediate, and high-risk groups is called risk stratification. It is important to have a platform to categorize patients according to their risk and take the initiative to successfully manage health for each population. Risk stratification is important for any disease, especially critical diseases. For heart disease, risk assessment in primary and secondary prevention enables decisions about the best treatment, lifestyle, medical and interventional care. For example, stabilizing the risk of acute myocardial infarction in patients is a prerequisite for improving patient-friendly treatments and prognosis. Biomarkers could be very useful in achieving this goal, as they can provide easily understood objective information [88]. Gagno et al. (2019) studied a total of 469 patients and found an average Gal-3BP value of 9.1 g/ml and an average Gal-3 value of 9.8 ng/ml. During the 12-month follow-up period, a total of 34 patients died, and 41 patients experienced angina/infarction. For all causes of death, G3BP is associated with an increased risk of angina/heart attack after adjusting for other important covariates [88].

The final multivariable models predicting death included patient age, left ventricular ejection fraction (LVEF), Gal-3, and renal function. The estimated value at the ROC curve in this model was 0.84 (95% CI 0.78–0.9), similar to the GRACE score for 1-year mortality. GRACE score is a scoring system used in patients with acute coronary syndromes to estimate in-hospital mortality in these patients. It is usually calculated for the6 months to 3-year period (https://www.mdcalc.com/grace-acs-risk-mortality-calculator). A comprehensive assessment of Gal-3 and Gal-3BP may help stabilize risk after acute myocardial infarction [88].

## Pathway and interactions study

**Fig. 4:**
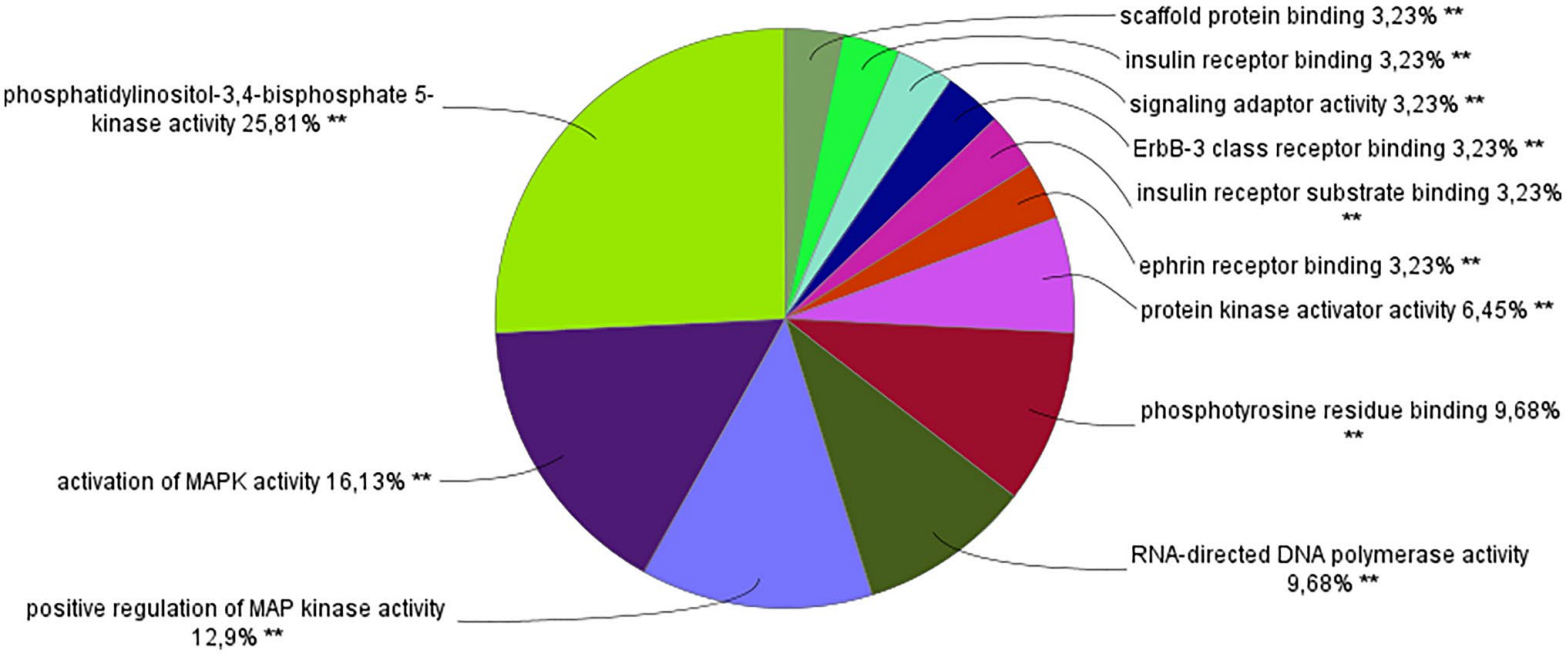
Pie chart of the percentage of terms per group on the ClueGo + CluePedia Cytoscape analysis of a list of proteins related/that interact with Gal-3, or vice-versa

Using the string online bioinformatic tool (https://string-db.org/) we searched for a network of proteins associated with Gal-3. Using the following parameters: number of nodes = 51, number of edges = 285 and minimum required interaction score = 0.7, we obtained a list of 51 unique proteins that are in a network of interactions with Gal-3. Processing this list of proteins with the Cytoscape bioinformatic tool using the ClueGo + CluePedia application, we obtained the results showed in Fig. 4. In this bioinformatic analysis, we found the following proteins involved in this process that interact with Gal-3: PIK3CA; PIK3CB; PIK3CG, PIK3R1 and SRC.

ClueGo + CluePedia analysis revealed that the following proteins have interaction with Gal-3 and MAPK: A-Raf, B-Raf, EGFT, ERBB3, MAPK1, MAP2K1, MAP2K2, MAPK3, H-Ras, K-Ras, Raf1, NF1, PIK3CA, PIK3CB, PIK3CG, PTPN11, RasGRP1, SOS1, SRC and SYK. The interactions between phosphatidylinositol-4,5-Bisphosphate 3-Kinase (PI3K) and mitogen-activated protein kinase (MAPK) account for more than 50% of the biological functions revealed by the Cytoscape bioinformatic tool. MAPK is a type of protein kinase involved in cellular responses such as osmotic stress and proinflammatory cytokines. MAPK is also involved in cell regulatory functions such as proliferation, differentiation, mitosis, apoptosis and gene expression [99]. PI3K is involved in processes such as: cell growth, cell proliferation, cell survival and glucose uptake [100]. The other processes such as telomerase activity, protein binding and polymerase activity are inherently related with cell division and proliferation, further supporting the previously mentioned findings.

## Conclusion

This review represents an attempt to draw attention to Gal-3 as an important player in two critical diseases, namely heart diseases and cancer with particular emphasis on PCa. Gal-3 can be analyzed and specific clinical pathogens, diagnostic and/or prognostic implications for Gal-3 have been suggested. The involvement of this protein in additional diseases needs to be analyzed so that it can be used as a suitable biomarker for them. The introduction and clinical application of Gal-3 inhibitors/antagonists strongly suggests that the accumulation of experimental and clinical evidence may open entirely new avenues for the treatment of skin inflammation and neoplastic diseases (psoriasis, atopic dermatitis, and melanoma). A comprehensive and in-depth understanding of the mechanism of its pathogenicity and the potential process of Gal-3 biological function may help in risk stratification, prognosis, and early diagnosis of life-threatening diseases.

As a multifunctional protein, Gal-3 is involved in many diseases. Its prognostic and diagnostic efficacy in cardiovascular, renal, autoimmune and cancer diseases is well established. The detection method for Gal-3 needs to be further developed to improve its sensitivity and accuracy, establish consensus among different laboratories, and supports its clinical application. In addition, normal reference limits need to be recognized. In addition, research on this protein in increasingly less invasive samples should be increased. Some steps in this direction have already been taken, such as the quantification of Gal-3 in the saliva of patients with HF and in the urine of patients with PCa, but more studies are needed.

Based on many of the previously mentioned studies, the relevance of Gal-3 in the clinical setting can be seen and how Gal-3-based clinical studies are rapidly being introduced into clinical practice to be used as diagnostic and/or prognostic biomarkers. Interestingly, almost all, if not all, information on the pathogenic role of Gal-3 in acute diseases should be translated into the clinical practice as soon as possible, as should its use in appropriate patients.

Currently, several clinical trials of Gal-3 are still ongoing in different areas: treatment of tumors, HF, fibrosis, metabolism, and degenerative diseases. Numerous pharmaceutical companies are investing in the development and testing of Gal-3 molecules. The extraordinary medical history of this lectin molecule is still in its early stages, but it is expected that a new era with new and exciting developments will be soon dawn.

## Declarations

### Research involving human and animals rights

This article does not contain any studies with human participants or animals performed by any of the authors.
